# Assessment of adherence level for neonatal hyperbilirubinemia management by various physicians in ‎Iraq: a multi-clinic study

**DOI:** 10.12688/f1000research.24258.1

**Published:** 2020-06-03

**Authors:** Numan Nafie Hameed, Hikmat Noori Yousif, Hayder Adnan Fawzi

**Affiliations:** 1 Department of Pediatrics‎, College of Medicine, Baghdad University, Baghdad, 12221, Iraq; 2 Department of Pediatrics‎, Children Welfare Teaching Hospital, Baghdad, 12221, Iraq; 3 College of Pharmacy, Al-Rasheed University College, Bagdad, 12221, Iraq

**Keywords:** adherence, neonatal hyperbilirubinemia, physicians, pediatrician‎

## Abstract

**Background:** Neonatal jaundice is a physiological process that occurs normally ‎for every infant to a varying degree. In some cases, this process becomes pathological ‎and imposes an increased risk of morbidity and mortality for the infant. The aim of this study was to assess the adherence level of various physicians to different guidelines ‎of management of neonatal hyperbilirubinemia in Iraq.

**Methods:** An observational cross-sectional study was conducted in ‎multiple outpatient clinics in various Iraqi provinces, from February ‎‎2018 to ‎February 2019. ‎The study involved 130 physicians, who were divided into emergency physicians (EPs), general practitioners (GPs), and pediatricians (PDs), and assessed their compliance to guidelines for management of neonatal hyperbilirubinemia using a questionnaire, which included providing the correct management for a test case scenario.

**Results:** PDs had significantly longer discharge times compared to EPs and GPs. In total, ‎91.7‎% of PDs always tested the neonate for bilirubin levels ‎before discharge, while 5.5‎% of GPs and 0% of EP did so. Regarding follow-up visits, 16.7‎%, 4.8% and 45.2% of PDs, EPs and GPs, respectively, scheduled a follow-up between 49-72 hours; ‎47.6‎% and 38.1% of EPs scheduled a follow-up at ≤24 hours and 25-48 hours, respectively‎‏. In addition, 91.7% of PDs gave the correct answer for the management of the test ‎case scenario, followed by 58.9% of GPs, and 38.1% of ‎EPs‎‏.‏ About half of ‎PDs extended neonates length of stay beyond 48 ‎hours.

**Conclusion:** GPs and EPs show lower adherence levels for the management of neonatal jaundice than PDs, which indicates that these physicians adhere well to current management guidelines from the WHO, ‎AAP, and NICE.

## Introduction

Hyperbilirubinemia (HBR) is one of the commonest causes of neonatal readmission to hospital ^1^, it requires urgent medical attention with rapid and prompt management to reduce bilirubin to prevent severe complications, such as kernicterus ^2^.

Despite the development of several guidelines (including the American Academy of Pediatrics (AAP) in 2004) to optimize the management of HBR, still the rate of inappropriate and insufficient therapy is rising ^3^, and consequently, the rate of morbidity will increase ^4^. These facts raise concerns about knowledge translation from and physician awareness of available guidelines in the literature and how much they use these in their daily practice ^5^.

Some surveys indicate that neonatologists are more aggressive than office-based general pediatricians in their treatment of neonatal jaundice in infants ^6^. While others show considerable variation in management depending on the specialty of the physician, with pediatricians being shown to have both exceptionally good awareness and adherence to guidelines. Thus, it is important to enhance knowledge translation to physicians, thereby increasing adherence to these guidelines, especially in physicians other than pediatricians ^5^.

Approximately 66% of pediatricians reported an awareness to neonatal hyperbilirubinemia clinical practice guidelines published in 1994 ^7^. However, Atkinson *et al* showed that only 54% of the pediatricians initiated treatment in accordance with recommended parameters ^8^. This study aimed to assess the level of adherence of various physicians to different guidelines of management of neonatal hyperbilirubinemia in a multi-clinic survey in Iraq.

## Methods

### Study design

This is an observational cross-sectional study conducted in multiple outpatient and private clinics in various Iraqi provinces. In this study, the investigator provided a questionnaire-based survey that assessed the adherence of various physicians to neonatal hyperbilirubinemia guidelines.

All procedures performed in the study were in accordance with the ethical standards of the Institutional Research Committee at the Children Welfare Teaching hospital, Baghdad City Complex, who approved the study protocol (approval date: 10 ^th^ January 2018; number: 2018/005), and with the 1964 Declaration of Helsinki and its later amendments. Written informed consent was obtained from all participants to be included in the study.

### Study setting

The study was conducted in various provinces in Iraq (Baghdad, Anbar, Diyala, and Nasiriya provinces) and targeted physicians both in private and public sectors. The period of recruitment was between February 2018 to February 2019. Hospitals at which the physicians were based were selected on whether or not the hospital had a neonatal care clinic. The participants were approached by the investigators at their place of work

### Sample size calculation

It is estimated that physician awareness about hyperbilirubinemia guideline is 70% according to the Christakis and Rivara study ^7^. Since this is a cross-sectional study, the following equation was used to calculate sample size:

$$N=\frac{4pq}{d^{2}}$$

N: sample size, p: prevalence, q = 100 – p, and d is precision (12%) d = (p/100) *x* p

A sample size of 120 was calculated. To account for a possible drop out of 20%, a sample of 144 physicians was proposed.

### Participants

Initially, we selected 144 physicians; 14 physicians did not participate in the study, leaving 130 physicians who completed the survey. The selection of physicians was based on whether they participated in the management of neonatal HBR. The physicians were divided according to their specialty: emergency physicians (EPs), general practitioners (GPs), and pediatricians (PDs).

### Data collection

Demographic data was collected from each physician (in an interview), including: age, years of practice, highest level of neonatal intensive care unit (NICU) the physician works in, and number of neonates per month seen by the physician.

A questionnaire ^9^ was used to assess the level of adherence to current guidelines. Questions included: length of hospital stay (discharge time) of neonates, physician policy about pre-discharge bilirubin level test, physician policy about timing of post-discharge follow-up, guideline used by the physician (WHO ^10^, AAP ^11^, and NICE ^12^), nature of guideline access, and problems encountered during management.

The questionnaire was reviewed and validated by three exports in medical education, neonatology, and biostatistics. After prior pilot study on 12 physicians, the reliability was calculated and found to be 0.89.

***Case scenario***. Additionally, a case scenario was presented to the physicians, as follows:

“A 30-year-old mother, with blood group O+, gave birth to a 2.8 kg male infant with cephalic hematoma after 37 weeks of gestation. Before discharge, at 36 hours after birth, the infant appeared jaundiced. I would do?”

The following options were provided to the physicians: discharge and follow-up, laboratory tests for bilirubin and blood group, cancel discharge and start phototherapy, refer to a pediatrician.

### Statistical analysis

Discrete variables were presented using their number and percentage. Chi-square test was used to analyze discrete variables (or Fisher exact test when chi-square was not valid; due to low sample size <20 and if 2 or more with an expected frequency was less than 5). SPSS 22.0.0 (Chicago, IL) software package was used to conduct statistical analysis. P-value of less than 0.05 was considered significant.

## Results

In total, 130 physicians participated in this questionnaire-based study: 21 (16.15%) were EPs, 73 (56.15%) GPs, and 36 (27.67%) PDs.

PDs had a significantly higher age compared to both ERs and GPs. The majority of all physician groups worked at level I NICU (66.7%, 72.6%, 50.0%; respectively); notably more PDs worked at level II and III NICU compared to other groups. Both GPs and PDs had a longer duration of practice compared to an EPs, and additionally, PDs saw the most neonates per month compared to EPs and GPs ( Table 1).

**Table 1.** Demographic characteristics of physicians.

	EP	GP	PD	P value
**N**	21	73	36	
**Age (years), n (%)**				0.004
*** < 30***	2 (9.5)	11 (15.1)	7 (19.4)	
*** 30 – 39***	14 (66.7)	24 (32.9)	10 (27.8)	
*** 40 – 49***	5 (23.8)	31 (42.5)	9 (25.0)	
*** 50 – 59***	0 (0)	7 (9.6)	10 (27.8)	
**Years of practice, n (%)**				<0.001
*** ≤ 1***	11 (52.4)	18 (24.7)	0 (0)	
*** 2 – 5***	4 (19.0)	36 (49.3)	24 (66.7)	
*** 6 – 9***	6 (28.6)	3 (4.1)	8 (22.2)	
*** ≥ 10***	0 (0)	16 (21.9)	4 (11.1)	
**Highest level of NICU in hospital practice,** **n (%)**				<0.001
*** Level I***	14 (66.7)	53 (72.6)	18 (50.0)	
*** Level II***	7 (33.3)	20 (27.4)	8 (22.2)	
*** Level III***	0 (0)	0 (0)	6 (16.7)	
*** Private***	0 (0)	0 (0)	4 (11.1)	
**No. of neonates seen per month, n (%)**				<0.001
*** ≤ 1***	5 (23.8)	27 (37.0)	0 (0)	
*** 2 – 5***	13 (61.9)	12 (16.4)	5 (13.9)	
*** 6 – 9***	3 (14.3)	18 (24.7)	8 (22.2)	
*** ≥ 10***	0 (0)	16 (21.9)	23 (63.9)	

ED, emergency physician; GP, general practitioner; PD, pediatrician. *Levels of NICU* ^13^: Level I: Well newborn nursery, Level II: Special care nursery, Level III: Neonatal intensive-care unit (NICU), Level IV: Regional neonatal intensive-care unit (Regional NICU).

PDs had significantly longer discharge times of neonates compared to EPs and GPs. In addition, 91.7% of PDs always tested the neonate for bilirubin levels before discharge, while only 5.5% of GPs and 0% of EPs did so. Regarding follow-up visits, 16.7%, 4.8% and 45.2% of PDs, EPs and GPs, respectively, scheduled a follow-up between 49–72 hours; 47.6% and 38.1% of EPs scheduled a follow-up at ≤24 hours and 25–48 hours, respectively ( Table 2).

**Table 2.** Assessment of physicians’ adherence to guidelines for treating neonates with hyperbilirubinemia.

	EP	GP	PD	P value
**N**	21	73	36	
**Discharge (length of stay; hours), n (%)**				<0.001
*** ≤ 24***	17 (81.0)	60 (82.2)	10 (27.8)	
*** 25 – 48***	3 (14.3)	13 (17.8)	5 (13.9)	
*** > 48 hrs***	1 (4.8)	0 (0)	21 (58.3)	
**Pre-discharge bilirubin level test, n (%)**				<0.001
*** Always***	0 (0)	4 (5.5)	33 (91.7)	
***Yes, if the baby*** ***looks jaundiced***	19 (90.5)	69 (94.5)	3 (8.3)	
*** No***	2 (9.5)	0 (0)	0 (0)	
**Timing of post-discharge follow-up (hours), n (%)**				<0.001
*** ≤24***	10 (47.6)	6 (8.2)	3 (8.3)	
*** 25 – 48***	8 (38.1)	28 (38.4)	24 (66.7)	
*** 49 – 72***	1 (4.8)	33 (45.2)	6 (16.7)	
*** >72***	2 (9.5)	6 (8.2)	3 (8.3)	
**Guideline used, n (%)**				<0.001
*** WHO***	6 (28.6)	15 (20.5)	4 (11.1)	
*** AAP***	2 (9.5)	1 (1.4)	31 (86.1)	
*** NICE***	0 (0)	3 (4.1)	1 (2.8)	
*** None***	13 (61.9)	54 (74.0)	0 (0)	
**Information access, n (%)**				<0.001
*** Books***	4 (19.0)	15 (20.5)	25 (69.4)	
*** Online***	1 (4.8)	6 (8.2)	11 (30.6)	
*** Notes***	1 (4.8)	0 (0)	0 (0)	
*** No***	15 (71.4)	52 (71.2)	0 (0)	
**Management of problems, n (%)**				
*** Education***	5 (23.8)	10 (13.7)	3 (8.3)	0.262
*** Diagnostic***	15 (71.4)	52 (71.2)	5 (13.9)	<0.001
*** Therapeutic***	6 (28.6)	48 (65.8)	8 (22.2)	<0.001
*** Facilities***	13 (61.9)	54 (74.0)	7 (19.4)	<0.001

ED, emergency physician; GP, general practitioner; PD, pediatrician.

Table 3 illustrates the assessment of physician knowledge toward assessment of HBR. It shows that recognizing visual signs and jaundice within 24 hours of birth was performed to a higher extent by PDs compared with EPs and GPs.

**Table 3.** Assessment of physicians’ knowledge for treating neonates with hyperbilirubinemia.

	EP	GP	PD	P-value
**N**	21	73	36	
***What are the warning signs of severe*** ***hyperbilirubinemia?***				
**Visual, n (%)**				0.013
*** Negative***	17 (81.0)	51 (69.9)	34 (94.4)	
*** Positive***	4 (19.0)	22 (30.1)	2 (5.6)	
**Jaundice <24 hours of birth, n (%)**				<0.001
*** Negative***	4 (19.0)	24 (32.9)	0 (0)	
*** Positive***	17 (81.0)	49 (67.1)	36 (100)	
**Jaundice within 1–14 days, the baby is active** **and growing, n (%)**				0.277
*** Negative***	20 (95.2)	68 (93.2)	36 (100)	
*** Positive***	1 (4.8)	5 (6.8)	0 (0)	
**Bilirubin level >10 mg/dL in term baby, n (%)**				1.0
*** Negative***	21 (100)	73 (100)	36 (100)	
*** Positive***	0 (0)	0 (0)	0 (0)	

ED, emergency physician; GP, general practitioner; PD, pediatrician.

In total, 91.7% of PDs gave the correct answer for the case scenario, followed by GPs (58.9%) and EPs (38.1%) ( Table 4).

**Table 4.** Assessment of physicians’ knowledge for treating neonates with hyperbilirubinemia using a case scenario.

	EP	GP	PD	P-value
**N**	21	73	36	
**Case scenario answers, n (%)**				0.002
*** Discharge and follow-up***	4 (19.0)	11 (15.1)	0 (0)	
*** Laboratory tests bilirubin and blood group***	8 (38.1)	43 (58.9)	33 (91.7)	
*** Cancel discharge and start phototherapy***	7 (33.3)	16 (21.9)	3 (8.3)	
*** Refer to pediatrician***	2 (9.5)	3 (4.1)	0 (0)	

ED, emergency physician; GP, general practitioner; PD, pediatrician. Correct answer: laboratory tests bilirubin and blood group.

## Discussion

In this survey, there was obvious variation in the adherence of various groups of physicians to guidelines for treating neonates with HRB. The results showed that 74% of GPs and 61.9% of EPs did not use any guidelines for the management and identification of neonatal HBR; PDs on the other hand did use guidelines. These findings were higher than reported in other countries. Sampurna *et al*, in a study conducted in Indonesia that involved 291 midwives, 206 GPs, and 154 PDs, reported that 23% of GPs and 29% of midwives were unaware of the presence of guidelines for identification and management of neonatal HBR ^14^. In a study by Mateo *et al*, which involved 321 Canadian physicians, they reported that guidelines were used by 41% of family physicians, 75% of PDs, and 69% of midwives, which is partially in agreement with our findings ^5^.

Early identification is the cornerstone of any guideline created to manage HBR ^3, 10, 15^. Monitoring of the neonate with HBR is achieved via measurement of bilirubin especially in the first 3 days of life ^16^, with subsequent follow-up every 24-48 hours till oral feeding is assured ^16^. In the present study, before discharge monitoring of bilirubin was being performed by only 5.5% of GPs, 0% of EPs and 91.7% of PDs. In addition, 90.5% of EPs and 94.5% of GPs reported the use of pre-discharge bilirubin testing only if the infant looked jaundiced. This finding is higher than that reported by a previous Iraqi study by Hameed and Abdul Razak ^17^, who reported that 56% of PDs did not consider post-discharge total serum bilirubin monitoring. In the Canadian study by Mateo *et al*, 42% of family physicians and 22% of midwives compared with 63% of PDs, reported bilirubin measurement before discharge, which is lower than reported by the current study ^5^. In the Indonesian study, 12% of GPs and 8% of PDs reported always performing pre-discharge bilirubin measurement, while 54% of GPs and 65% of PDs reported the use of pre-discharge bilirubin measurement only if jaundice was present, which is partially in agreement with our findings ^14^. In addition, in a study by Petrova *et al*, which involved 356 PDs in the USA, they reported that 87.4% of all physicians used TSB testing with clinical jaundice before discharge, while 57.7% of them only used TSB testing in clinical jaundice post-discharge, which in agreement with our findings ^18^.

In the present study, all PDs considered jaundice in less than 24 hours of life as an indicator of severe HBR, compared with 81.0% of EPs and 67.1% of GPs. This is in agreement with the study by Hameed and Abdul Razak that showed that 76% of PDs strongly agreed that neonates presenting with jaundice in the first 24 hours as a predictor of severe HBR ^17^. Petrova *et al* reported similar findings: 77% of physicians considered that jaundice presenting in the first 24 hours was a marker for severe HBR ^18^. Several studies in the literature indicate the importance of early first-day measurement of bilirubin levels since elevated levels are correlated strongly with later severe complications ^19– 21^. There was good knowledge of this by physicians in this study.

In the present study, PDs relied less on visual assessment of jaundice (5.6%), jaundice in stool and fever (0%), and jaundice at 14 days when the baby is active and growing (0%), compared to EDs and GPs. The Hameed and Abdul Razak study reported few PDs (10%) agreed that jaundice noticed at discharge is a risk factor for severe HBR ^17^, which was in agreement with our findings. Around 61% of primary health care provider assessed the severity of neonatal jaundice by visual cephalocaudal evaluation inspecting the skin or sclera, which is rapid and cost-free, but it is not sufficiently accurate, especially when applied to newborns with dark skin ^22^, which disagrees with our findings.

In the present study, the majority of PDs gives the correct answer (laboratory tests bilirubin and blood group) to the case scenario (91.7%); however lower correct answer rates were achieved by GPs (58.9%) and EPs (38.1%) (p-value = 0.002). In the Mateo *et al* study, the response to a case scenario similar to ours showed similar results to our findings, while in the Sampurna *et al* study, the response to a similar case scenario showed a lower correct answer rate with 54% of PDs, 57% of GPs, and 44% of midwives providing the correct answer ^14^.

Hospital affiliation of health care providers is one of the strongest predictors of adherence to practice guidelines ^6^, which is one of the possible explanations of the high rate of good knowledge about the management of neonatal HBR shown in the present study. The variability of the use of guidelines for the management of HBR in this study was similar to others ^8, 23^. Barriers to adherence include low awareness, no agreement, and inertia of previous practices ^24^.

### Study limitation

The study included only three types of physicians, other physicians that have role in the management of HBR were not included in the study. A nationwide study is required in the future to assess the overall adherence of physicians to the management of HBR. Another limitation is that we did not examine if the physicians in fact practiced what they had indicated in the survey.

## Conclusions

GPs and EPs in this study, performed in Iraqi hospitals, showed lower adherence levels for the management of neonatal jaundice than PDs, who exhibit excellent adherence to current guidelines (WHO, AAP, and NICE guidelines), especially AAP guidelines. We recommend starting an education program directed toward the enhancement of knowledge for EPs and GPs for the management of neonatal jaundice.

## Data availability

Zenodo: Adherence Level for Neonatal Hyperbilirubinemia, https://doi.org/10.5281/zenodo.3745611 ^25^.

### Extended data

Zenodo: Questionnaire about Hyperbilirubinemia, http://doi.org/10.5281/zenodo.3860692 ^9^

Data are available under the terms of the Creative Commons Attribution 4.0 International license (CC-BY 4.0).
