# Supplementary material for: Assessment of adherence level for neonatal hyperbilirubinemia management by various physicians in ‎Iraq: a multi-clinic study
Source: F1000Res. 2020 Jun 3;9:504. [Version 1] doi: 10.12688/f1000research.24258.1 (PMC7388196; doi:10.12688/f1000research.24258.1)
Supplement: Peer review history

# Peer review: Reviewer response for version 1

**DOI:** 10.5256/f1000research.26761.r65857

**Published:** 2020-07-28

**Authors:** Laila M. Sherief

**Affiliations:** 1 Pediatric Oncology Unit, Faculty of Medicine, Zagazig University, Zagazig, Egypt

This manuscript assesses the adherence of various physicians to guidelines to important and sometimes serious problems in the neonate.

 The methods: I want to ask the authors, why ER doctors included in the work

 Results: Table 3: need more clarification.

 Discussion: needs to be deeper with more explanation of the differences between the results

Is the work clearly and accurately presented and does it cite the current literature?

Yes

If applicable, is the statistical analysis and its interpretation appropriate?

I cannot comment. A qualified statistician is required.

Are all the source data underlying the results available to ensure full reproducibility?

Yes

Is the study design appropriate and is the work technically sound?

Yes

Are the conclusions drawn adequately supported by the results?

Yes

Are sufficient details of methods and analysis provided to allow replication by others?

Yes

Reviewer Expertise:

pediatric hematology and oncology and neonatology

I confirm that I have read this submission and believe that I have an appropriate level of expertise to confirm that it is of an acceptable scientific standard.

## References

[1] : Knowledge of Neonatal Hyperbilirubinemia Among Primary Health Care Physicians: A Single-Center Experience. *Clin Med Insights Pediatr*.2019;13: 10.1177/1179556518824375 1179556518824375 10.1177/1179556518824375 30853790PMC6399760

# Peer review: Reviewer response for version 1

**DOI:** 10.5256/f1000research.26761.r64296

**Published:** 2020-06-10

**Authors:** Robert D. Christensen

**Affiliations:** 1 Division of Neonatology and Hematology/Oncology, University of Utah Health, Salt Lake City, UT, USA

The submission by Dr. Hameed *et al.* reviews results of a questionnaire completed by 130 physicians in Iraq, about neonatal bilirubin management. The authors are to be congratulated on this study, analysis, and report. Perhaps they would consider the following somewhat minor issues, all aimed at improving the clarity of the report.

1. Title: Consider deleting the phrase “adherence level for”. It suggests that there are different levels. The title is clearer without that phrase.
2. Abstract: It is not until the last line of the Abstract Conclusion that we read the guidelines of interest are those of the WHO, AAP, and NICE. Placing this near the beginning of the abstract would be helpful. Also, under Abstract Methods, consider changing …”physicians, who were divided into…”  to, “physicians self-identified as either…”. Also, under Abstract Conclusions, delete “adherence levels”, because various levels of adherence was not part of the analysis.
3. Introduction: Second line. Instead of, “…to hospital, it requires urgent”, consider, “to hospital, severe cases require…”. Last paragraph before ***Case Scenario,*** *change “exports” to “experts”.*
4. Results: In Table 3, it is not clear what the “negative” and “positive” indicates. Thus, I can not understand what is being conveyed by this table.

Is the work clearly and accurately presented and does it cite the current literature?

Yes

If applicable, is the statistical analysis and its interpretation appropriate?

Yes

Are all the source data underlying the results available to ensure full reproducibility?

Yes

Is the study design appropriate and is the work technically sound?

Yes

Are the conclusions drawn adequately supported by the results?

Yes

Are sufficient details of methods and analysis provided to allow replication by others?

Yes

Reviewer Expertise:

Neonatal Hematology

I confirm that I have read this submission and believe that I have an appropriate level of expertise to confirm that it is of an acceptable scientific standard.
